# Laser lithotripsy for refractory stones in the cystic duct and common bile duct

**DOI:** 10.1055/a-2767-0801

**Published:** 2026-01-15

**Authors:** Haruo Miwa, Yugo Ishino, Kazuki Endo, Ritsuko Oishi, Yuichi Suzuki, Hiromi Tsuchiya, Shin Maeda

**Affiliations:** 1 26437 Gastroenterological Center, Yokohama City University Medical Center, Yokohama, Kanagawa, Japan; 2 Department of Gastroenterology, Yokohama City University Graduate School of Medicine, Yokohama, Kanagawa, Japan

Mirizzi syndrome caused by multiple cystic duct (CD) stones represents a challenging condition for endoscopic management 1 . Although peroral cholangioscopy (POCS)-guided lithotripsy is effective 2 3 , the optimal energy source, electrohydraulic lithotripsy (EHL) or laser lithotripsy remains undetermined 4 . Recently, a new Holimium:YAG-laser system (LithoEVO; Edap TMS, Lyon, France) has become available, offering enhanced energy control 5 .

**Fig. 1:**
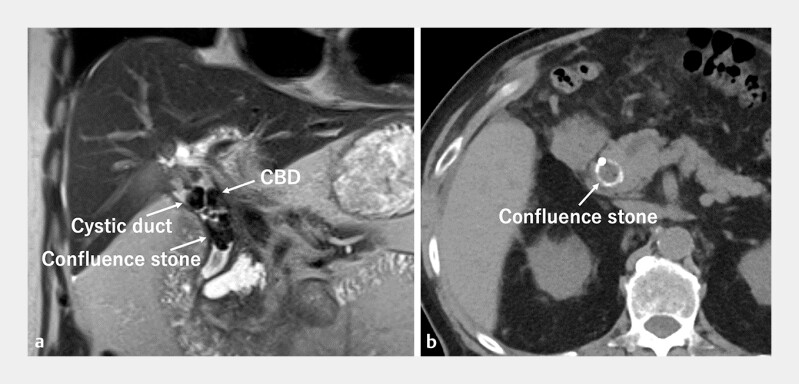
Images of biliary stones. **a** A magnetic resonance image shows a huge confluence stone and multiple stones in the cystic duct and common bile duct. **b** A computed tomographic image shows a calcified confluence stone.

**Fig. 2:**
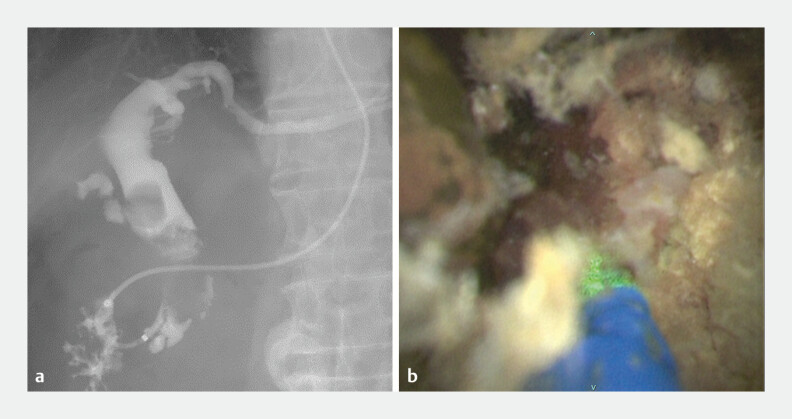
First session of cholangioscopy-guided laser lithotripsy. **a** Cholangiography shows a huge confluence stone and multiple cystic duct stones. **b** Laser lithotripsy was effective for cystic duct stones.

**Fig. 3:**
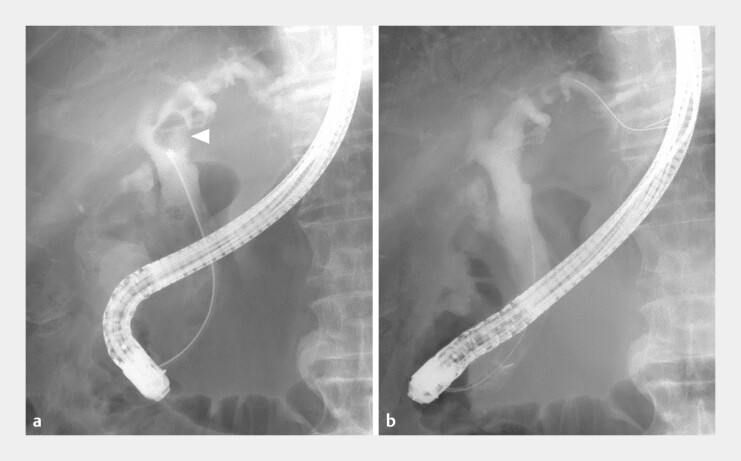
Second session of cholangioscopy-guided laser lithotripsy. **a** A large stone in the perihilar bile duct (Arrowhead) was effectively crushed. **b** Cholangiography after stone retrieval shows complete stone clearance.

An 84-year-old man was referred to our hospital with cholangitis due to multiple stones in the CD and common bile duct (CBD; Fig. 1 ). POCS-guided lithotripsy with EHL failed to fragment the hard stone in the CD, even after three attempts. Because the patient declined surgical intervention, stent placement was performed; however, recurrent cholangitis occurred. Therefore, POCS-guided laser lithotripsy was undertaken ( Video 1 ). During the first session, a 9-Fr eyeMAX cholangioscope (Micro-Tech, Nanjing, China) was advanced into the cystic duct. Although the cystic duct stones were extremely hard, effective fragmentation was achieved by increasing both laser frequency from 10 to 15 Hz and energy from 1.0 to 1.2 J. The characteristic green aiming beam enabled safe and precise targeting, even when the visualization was obscured by stone fragments filling the CD ( Fig. 2 ). The crushed stones were subsequently retrieved using a mechanical lithotripter (Stone Smash; Boston Scientific Japan, Tokyo, Japan). In the second session, the stones in the CBD were crushed. A large and hard perihilar stone was successfully disintegrated with laser lithotripsy, followed by stone extraction using the lithotripter and a retrieval basket (8-wire basket, Medi-Globe, Germany; Fig. 3 ). After two sessions, the completer clearance of both the CD and CBD stones was confirmed with cholangioscopy. The patient was discharged without any complications.

This case demonstrates that laser lithotripsy using a novel Holmium: YAG laser system can achieve safe and effective stone clearance in refractory Mirizzi syndrome with multiple stones in the CD and CBD.

Laser lithotripsy using a novel Holmium:YAG laser system for refractory Mirizzi syndrome with multiple stones in the CD and CBD. CD, cystic duct; CBD, common bile duct.Video 1

Endoscopy_UCTN_Code_TTT_1AR_2AH
